# 2-(2-Amino-4-nitro­phen­yl)-7-nitro-4*H*-3,1-benzoxazin-4-one

**DOI:** 10.1107/S1600536814000609

**Published:** 2014-01-18

**Authors:** Edward R. T. Tiekink, James L. Wardell

**Affiliations:** a Department of Chemistry, University of Malaya, 50603 Kuala Lumpur, Malaysia; b Fundação Oswaldo Cruz, Instituto de Tecnologia em, Fármacos–Farmanguinhos, R. Sizenando Nabuco, 100, Manguinhos 21041-250, Rio de Janeiro, RJ, Brazil; c Chemistry Department, University of Aberdeen, Old Aberdeen AB24 3UE, Scotland

## Abstract

In the title compound, C_14_H_8_N_4_O_6_, the benzoxazin-4-one fused-ring system (r.m.s. deviation = 0.018 Å) is coplanar with the attached benzene ring [dihedral angle = 0.81 (4)°], there being an intra­molecular N—H⋯N hydrogen bond between them. Each nitro group is twisted out of the plane of the attached benzene ring [O—N—C—C torsion angles = 167.94 (11) and 170.38 (11)°]. In the crystal, amine–nitro N—H⋯O hydrogen bonds lead to centrosymmetric dimeric aggregates that are connected into a three-dimensional architecture by oxazin­yl–nitro C—H⋯O and π–π inter­actions [inter-centroid distance between the oxazinyl and terminal benzene rings = 3.5069 (7) Å].

## Related literature   

For background to the spectroscopic characteristics of *N*-derivatives of 2-(2-amino­phen­yl)-4*H*-3,1-benzoxazin-4-ones, see: Loseva *et al.* (1971 ▶,1972 ▶); Eberius & Hügin (2012 ▶); Khimich *et al.* (2009 ▶, 2010 ▶). For their synthesis, see: Bolotin *et al.* (1965 ▶); Brudz *et al.* (1967 ▶); Loseva *et al.* (1971 ▶, 1972 ▶); Eberius & Hügin (2012 ▶).
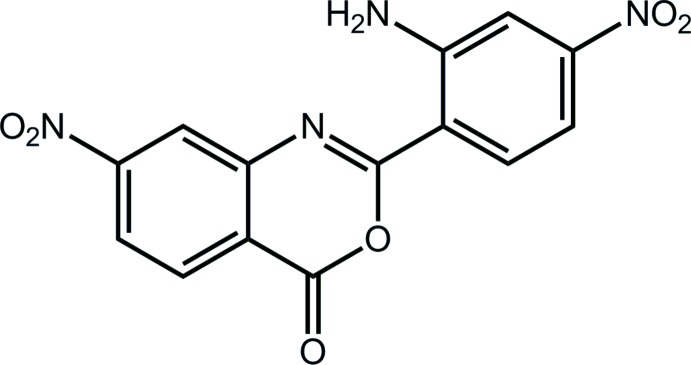


## Experimental   

### 

#### Crystal data  

C_14_H_8_N_4_O_6_
*M* *_r_* = 328.24
Monoclinic, 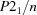

*a* = 7.0229 (3) Å
*b* = 8.6148 (3) Å
*c* = 21.5662 (15) Å
β = 90.029 (6)°
*V* = 1304.77 (12) Å^3^
*Z* = 4
Mo *K*α radiation
μ = 0.14 mm^−1^
*T* = 100 K
0.13 × 0.08 × 0.03 mm

#### Data collection  

Rigaku R-AXIS conversion diffractometer
Absorption correction: multi-scan (*CrystalClear-SM Expert*; Rigaku, 2012 ▶) *T*_min_ = 0.831, *T*_max_ = 1.000
16840 measured reflections
2983 independent reflections
2513 reflections with *I* > 2σ(*I*)
*R*_int_ = 0.026

#### Refinement  

*R*[*F* ^2^ > 2σ(*F* ^2^)] = 0.034
*wR*(*F* ^2^) = 0.101
*S* = 1.12
2983 reflections
223 parameters
2 restraints
H atoms treated by a mixture of independent and constrained refinement
Δρ_max_ = 0.38 e Å^−3^
Δρ_min_ = −0.21 e Å^−3^

### 

Data collection: *CrystalClear-SM Expert* (Rigaku, 2012 ▶); cell refinement: *CrystalClear-SM Expert*; data reduction: *CrystalClear-SM Expert*; program(s) used to solve structure: *SHELXS97* (Sheldrick, 2008 ▶); program(s) used to refine structure: *SHELXL97* (Sheldrick, 2008 ▶); molecular graphics: *ORTEP-3 for Windows* (Farrugia, 2012 ▶) and *DIAMOND* (Brandenburg, 2006 ▶); software used to prepare material for publication: *publCIF* (Westrip, 2010 ▶).

## Supplementary Material

Crystal structure: contains datablock(s) general, I. DOI: 10.1107/S1600536814000609/hg5375sup1.cif

Structure factors: contains datablock(s) I. DOI: 10.1107/S1600536814000609/hg5375Isup2.hkl

Supporting information file. DOI: 10.1107/S1600536814000609/hg5375Isup3.cml

CCDC reference:

Additional supporting information:  crystallographic information; 3D view; checkCIF report

## Figures and Tables

**Table 1:** Hydrogen-bond geometry (Å, °)

*D*—H⋯*A*	*D*—H	H⋯*A*	*D*⋯*A*	*D*—H⋯*A*
N2—H1*N*⋯N3	0.89 (1)	2.06 (1)	2.7124 (14)	130 (1)
N2—H2*N*⋯O1^i^	0.89 (1)	2.20 (1)	3.0691 (14)	166 (1)
C11—H11⋯O5^ii^	0.95	2.48	3.3248 (14)	149
C13—H13⋯O4^iii^	0.95	2.38	3.1778 (14)	141

Symmetry codes: (i) 
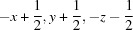
; (ii) 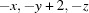; (iii) 
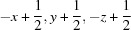
.

**Fig. 1.**
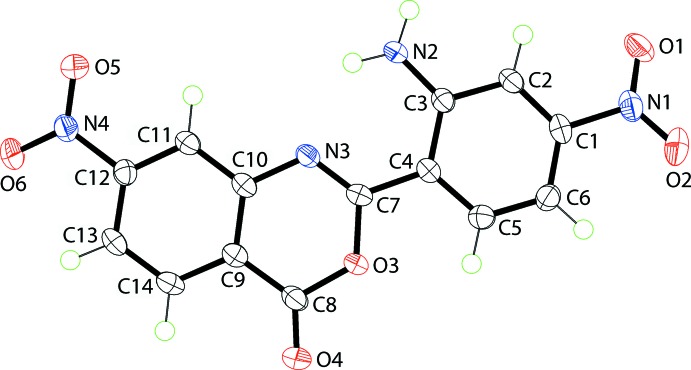
The molecular structure of (I) showing the atom-labelling scheme and displacement ellipsoids at the 70% probability level.

**Fig. 2.**
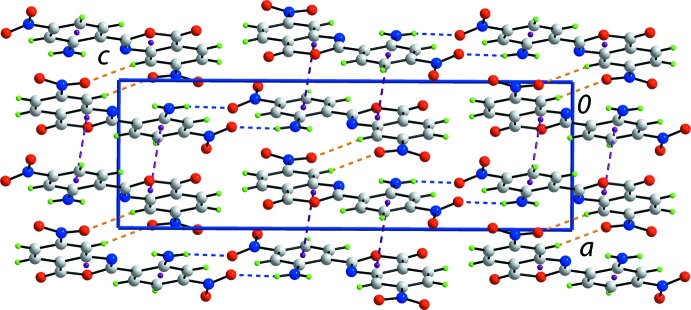
A view in projection down the b axis of the unit-cell contents for (I). The N—H···O, C—H···O and π—π interactions are shown as blue, orange and purple dashed lines, respectively.

## supplementary crystallographic information

### 2.1. Synthesis and crystallization

The title compound was obtained from the reaction of 2-amino-4-nitro­benzoic acid with 4-chloro­benzene­sulfonyl chloride (1 mmol of each) in refluxing acetone (20 ml) for 30 min. The reaction mixture was rotary evaporated and the residue was recrystallized from MeOCH_2_CH_2_OH. Crystals used in the structure determination were grown by slow evaporation of its MeOCH_2_CH_2_OH solution; M.pt: 474–475 K (dec.).

### 3. Refinement

Intensity data was collected at the National Crystallographic Service, England (Coles & Gale, 2012). The C-bound H atoms were geometrically placed (C—H = 0.95 Å) and refined as riding with *U*_iso_(H) = 1.2*U*_eq_(C). The N-bound H atoms were located from a difference map and refined with N—H = 0.88±0.01 Å, and with *U*_iso_(H) = 1.2*U*_eq_(N).

### 4. Results and discussion

The luminescent (Loseva *et al.*, 1971; Loseva *et al.*, 1972), fluorescent (Eberius & Hügin, 2012) and intra­molecular photoinduced proton transfer (IPPT) properties (Khimich *et al.*, 2009, 2010) of *N*-derivatives of 2-(2-amino­phenyl)-4*H*-3,1-benzoxazin-4-ones have attracted much attention. IPPT plays an important role in both chemical and biological processes (Khimich *et al.*, 2009; Khimich *et al.*, 2010 and references therein). The spectral properties are influenced by the strength of the intra­molecular N—H···N hydrogen bond. Generally, the preparation of 2-(2-amino­phenyl)-4H-3,1-benzoxazin-4-ones involves a dimerization of an anthranilic acid derivative in the presence of SO_2_Cl_2_ (Brudz *et al.*, 1967; Eberius & Hügin, 2012), arene­sulfonyl chloride (Loseva *et al.*, 1971; Loseva *et al.*, 1972) or PhCH_2_Cl (Bolotin *et al.*, 1965) in pyridine. Herein, the crystal and molecular structure of the title compound, (I), is described.

In (I), Fig. 1, the atoms comprising the benzoxazin-4-one fused-ring system are co-planar (r.m.s. deviation = 0.018 Å) and form a dihedral angle of 0.81 (4)° with the attached benzene ring. The co-planarity between the ring systems is accompanied by an intra­molecular N2—H···N3 hydrogen bond, Table 1. Both nitro groups are twisted out of the plane of the attached benzene rings as seen in the values of the O1—N1—C1—C2 and O6—N4—C12—C11 torsion angles of 167.94 (11) and 170.38 (11)°, respectively.

In the crystal packing, centrosymmetric dimeric aggregates are formed *via* 14-membered {···HNC_3_NO}_2_ synthons featuring amine-N—H···O(nitro) hydrogen bonds. These are connected into a three-dimensional architecture by oxazinyl-C—H···O(nitro) inter­actions as well as by π—π inter­actions between the oxazinyl and terminal benzene rings [inter­centroid distance = 3.5069 (7) Å, inter­planar angle = 1.11 (5)° for symmetry operation: 1-*x*, 1-*y*, -*z*].

### Crystal data

**Table:**

C_14_H_8_N_4_O_6_	*F*(000) = 672
*M**_r_* = 328.24	*D*_x_ = 1.671 Mg m^−^^3^
Monoclinic, *P*2_1_/*n*	Mo *K*α radiation, λ = 0.71073 Å
Hall symbol: -P 2yn	Cell parameters from 14670 reflections
*a* = 7.0229 (3) Å	θ = 3.0–27.5°
*b* = 8.6148 (3) Å	µ = 0.14 mm^−^^1^
*c* = 21.5662 (15) Å	*T* = 100 K
β = 90.029 (6)°	Plate, yellow
*V* = 1304.77 (12) Å^3^	0.13 × 0.08 × 0.03 mm
*Z* = 4	

### Data collection

**Table:**

Rigaku R-AXIS conversion diffractometer	2983 independent reflections
Radiation source: Sealed Tube	2513 reflections with *I* > 2σ(*I*)
Graphite monochromator	*R*_int_ = 0.026
Detector resolution: 10.0000 pixels mm^-1^	θ_max_ = 27.5°, θ_min_ = 3.0°
profile data from ω–scans	*h* = −9→9
Absorption correction: multi-scan (*CrystalClear-SM Expert*; Rigaku, 2012)	*k* = −10→11
*T*_min_ = 0.831, *T*_max_ = 1.000	*l* = −28→28
16840 measured reflections	

### Refinement

**Table:**

Refinement on *F*^2^	Primary atom site location: structure-invariant direct methods
Least-squares matrix: full	Secondary atom site location: difference Fourier map
*R*[*F*^2^ > 2σ(*F*^2^)] = 0.034	Hydrogen site location: inferred from neighbouring sites
*wR*(*F*^2^) = 0.101	H atoms treated by a mixture of independent and constrained refinement
*S* = 1.12	*w* = 1/[σ^2^(*F*_o_^2^) + (0.0557*P*)^2^ + 0.2657*P*] where *P* = (*F*_o_^2^ + 2*F*_c_^2^)/3
2983 reflections	(Δ/σ)_max_ = 0.001
223 parameters	Δρ_max_ = 0.38 e Å^−^^3^
2 restraints	Δρ_min_ = −0.21 e Å^−^^3^

### Special details

**Table:**

Geometry. All s.u.'s (except the s.u. in the dihedral angle between two l.s. planes) are estimated using the full covariance matrix. The cell s.u.'s are taken into account individually in the estimation of s.u.'s in distances, angles and torsion angles; correlations between s.u.'s in cell parameters are only used when they are defined by crystal symmetry. An approximate (isotropic) treatment of cell s.u.'s is used for estimating s.u.'s involving l.s. planes.
Refinement. Refinement of *F*^2^ against ALL reflections. The weighted *R*-factor *wR* and goodness of fit *S* are based on *F*^2^, conventional *R*-factors *R* are based on *F*, with *F* set to zero for negative *F*^2^. The threshold expression of *F*^2^ > 2σ(*F*^2^) is used only for calculating *R*-factors(gt) *etc*. and is not relevant to the choice of reflections for refinement. *R*-factors based on *F*^2^ are statistically about twice as large as those based on *F*, and *R*- factors based on ALL data will be even larger.

### Fractional atomic coordinates and isotropic or equivalent isotropic displacement parameters (Å^2^)

**Table:**

	*x*	*y*	*z*	*U*_iso_*/*U*_eq_	
O1	0.31373 (16)	0.12873 (11)	−0.24884 (4)	0.0331 (3)	
O2	0.46051 (15)	−0.04287 (11)	−0.19338 (5)	0.0346 (3)	
O3	0.31958 (12)	0.37682 (9)	0.06796 (4)	0.01839 (19)	
O4	0.33686 (15)	0.36166 (10)	0.17002 (4)	0.0293 (2)	
O5	0.01987 (14)	1.12855 (10)	0.07508 (4)	0.0255 (2)	
O6	−0.00939 (15)	1.12189 (10)	0.17491 (4)	0.0305 (2)	
N1	0.37678 (15)	0.08006 (12)	−0.19950 (5)	0.0217 (2)	
N2	0.19120 (16)	0.56949 (12)	−0.11084 (5)	0.0200 (2)	
H1N	0.175 (2)	0.6344 (14)	−0.0794 (5)	0.024*	
H2N	0.176 (2)	0.5998 (16)	−0.1498 (5)	0.024*	
N3	0.21171 (14)	0.59740 (11)	0.01426 (4)	0.0160 (2)	
N4	0.03554 (15)	1.06317 (12)	0.12548 (4)	0.0193 (2)	
C1	0.34836 (16)	0.17765 (13)	−0.14391 (5)	0.0174 (2)	
C2	0.28728 (16)	0.32695 (13)	−0.15271 (5)	0.0169 (2)	
H2	0.2653	0.3648	−0.1935	0.020*	
C3	0.25693 (16)	0.42466 (13)	−0.10088 (5)	0.0154 (2)	
C4	0.29441 (16)	0.36214 (13)	−0.04081 (5)	0.0156 (2)	
C5	0.35508 (16)	0.20706 (13)	−0.03512 (5)	0.0182 (2)	
H5	0.3774	0.1660	0.0051	0.022*	
C6	0.38315 (16)	0.11279 (13)	−0.08591 (5)	0.0185 (2)	
H6	0.4243	0.0083	−0.0815	0.022*	
C7	0.27046 (16)	0.45587 (13)	0.01513 (5)	0.0155 (2)	
C8	0.29892 (17)	0.44138 (14)	0.12669 (5)	0.0192 (2)	
C9	0.23325 (16)	0.60264 (13)	0.12738 (5)	0.0165 (2)	
C10	0.19409 (16)	0.67456 (13)	0.07059 (5)	0.0154 (2)	
C11	0.13195 (16)	0.82896 (13)	0.06998 (5)	0.0162 (2)	
H11	0.1066	0.8811	0.0321	0.019*	
C12	0.10881 (16)	0.90273 (13)	0.12612 (5)	0.0163 (2)	
C13	0.14479 (17)	0.83313 (14)	0.18319 (5)	0.0181 (2)	
H13	0.1260	0.8882	0.2208	0.022*	
C14	0.20854 (17)	0.68167 (14)	0.18338 (5)	0.0186 (2)	
H14	0.2356	0.6312	0.2215	0.022*	

### Atomic displacement parameters (Å^2^)

**Table:**

	*U*^11^	*U*^22^	*U*^33^	*U*^12^	*U*^13^	*U*^23^
O1	0.0600 (7)	0.0259 (5)	0.0136 (4)	−0.0009 (4)	0.0021 (4)	−0.0027 (3)
O2	0.0458 (6)	0.0254 (5)	0.0328 (5)	0.0117 (4)	0.0024 (4)	−0.0102 (4)
O3	0.0264 (5)	0.0176 (4)	0.0111 (4)	0.0030 (3)	−0.0010 (3)	0.0008 (3)
O4	0.0483 (6)	0.0254 (5)	0.0143 (4)	0.0105 (4)	−0.0021 (4)	0.0030 (3)
O5	0.0398 (5)	0.0188 (4)	0.0180 (4)	0.0026 (4)	0.0014 (4)	0.0024 (3)
O6	0.0496 (6)	0.0241 (5)	0.0178 (5)	0.0081 (4)	0.0070 (4)	−0.0055 (3)
N1	0.0260 (6)	0.0192 (5)	0.0199 (5)	−0.0028 (4)	0.0055 (4)	−0.0049 (4)
N2	0.0319 (6)	0.0173 (5)	0.0107 (5)	0.0028 (4)	−0.0007 (4)	0.0005 (4)
N3	0.0188 (5)	0.0175 (5)	0.0116 (4)	−0.0004 (4)	0.0002 (4)	−0.0002 (4)
N4	0.0239 (5)	0.0180 (5)	0.0161 (5)	−0.0006 (4)	0.0021 (4)	−0.0024 (4)
C1	0.0166 (5)	0.0192 (5)	0.0164 (5)	−0.0023 (4)	0.0026 (4)	−0.0053 (4)
C2	0.0192 (6)	0.0192 (5)	0.0122 (5)	−0.0017 (4)	0.0012 (4)	−0.0004 (4)
C3	0.0164 (5)	0.0162 (5)	0.0136 (5)	−0.0016 (4)	0.0013 (4)	−0.0008 (4)
C4	0.0161 (5)	0.0179 (5)	0.0129 (5)	−0.0005 (4)	0.0003 (4)	−0.0010 (4)
C5	0.0189 (6)	0.0191 (6)	0.0165 (5)	0.0009 (4)	−0.0013 (4)	0.0012 (4)
C6	0.0189 (6)	0.0164 (5)	0.0202 (6)	0.0020 (4)	−0.0004 (4)	−0.0006 (4)
C7	0.0157 (5)	0.0188 (5)	0.0121 (5)	−0.0013 (4)	−0.0011 (4)	0.0016 (4)
C8	0.0226 (6)	0.0229 (6)	0.0121 (5)	0.0007 (5)	0.0001 (4)	−0.0005 (4)
C9	0.0169 (6)	0.0192 (6)	0.0133 (5)	−0.0004 (4)	0.0006 (4)	0.0004 (4)
C10	0.0151 (5)	0.0189 (5)	0.0123 (5)	−0.0020 (4)	0.0007 (4)	−0.0006 (4)
C11	0.0176 (6)	0.0185 (5)	0.0126 (5)	−0.0014 (4)	0.0007 (4)	0.0005 (4)
C12	0.0169 (6)	0.0157 (5)	0.0162 (5)	−0.0015 (4)	0.0010 (4)	−0.0016 (4)
C13	0.0196 (6)	0.0231 (6)	0.0116 (5)	−0.0018 (4)	0.0018 (4)	−0.0027 (4)
C14	0.0202 (6)	0.0237 (6)	0.0118 (5)	0.0003 (4)	−0.0008 (4)	0.0018 (4)

### Geometric parameters (Å, º)

**Table:**

O1—N1	1.2263 (14)	C2—H2	0.9500
O2—N1	1.2185 (14)	C3—C4	1.4273 (15)
O3—C7	1.3712 (13)	C4—C5	1.4076 (16)
O3—C8	1.3910 (13)	C4—C7	1.4615 (15)
O4—C8	1.1897 (14)	C5—C6	1.3779 (16)
O5—N4	1.2291 (13)	C5—H5	0.9500
O6—N4	1.2215 (13)	C6—H6	0.9500
N1—C1	1.4780 (14)	C8—C9	1.4639 (16)
N2—C3	1.3476 (15)	C9—C14	1.3972 (15)
N2—H1N	0.886 (9)	C9—C10	1.3997 (15)
N2—H2N	0.886 (9)	C10—C11	1.3999 (16)
N3—C7	1.2873 (15)	C11—C12	1.3771 (15)
N3—C10	1.3904 (14)	C11—H11	0.9500
N4—C12	1.4749 (15)	C12—C13	1.3921 (16)
C1—C2	1.3690 (16)	C13—C14	1.3795 (17)
C1—C6	1.3913 (16)	C13—H13	0.9500
C2—C3	1.4156 (15)	C14—H14	0.9500
			
C7—O3—C8	122.12 (9)	C5—C6—H6	121.5
O2—N1—O1	124.42 (10)	C1—C6—H6	121.5
O2—N1—C1	118.16 (10)	N3—C7—O3	124.26 (10)
O1—N1—C1	117.42 (10)	N3—C7—C4	123.25 (10)
C3—N2—H1N	120.5 (10)	O3—C7—C4	112.49 (9)
C3—N2—H2N	117.6 (10)	O4—C8—O3	117.45 (11)
H1N—N2—H2N	121.6 (14)	O4—C8—C9	127.60 (11)
C7—N3—C10	117.96 (9)	O3—C8—C9	114.95 (9)
O6—N4—O5	123.98 (10)	C14—C9—C10	121.07 (10)
O6—N4—C12	118.05 (9)	C14—C9—C8	120.70 (10)
O5—N4—C12	117.95 (9)	C10—C9—C8	118.22 (10)
C2—C1—C6	123.84 (10)	N3—C10—C9	122.37 (10)
C2—C1—N1	117.67 (10)	N3—C10—C11	118.28 (10)
C6—C1—N1	118.49 (10)	C9—C10—C11	119.34 (10)
C1—C2—C3	119.76 (10)	C12—C11—C10	117.84 (10)
C1—C2—H2	120.1	C12—C11—H11	121.1
C3—C2—H2	120.1	C10—C11—H11	121.1
N2—C3—C2	118.45 (10)	C11—C12—C13	123.86 (11)
N2—C3—C4	123.86 (10)	C11—C12—N4	117.75 (10)
C2—C3—C4	117.68 (10)	C13—C12—N4	118.36 (10)
C5—C4—C3	119.54 (10)	C14—C13—C12	117.96 (10)
C5—C4—C7	119.16 (10)	C14—C13—H13	121.0
C3—C4—C7	121.30 (10)	C12—C13—H13	121.0
C6—C5—C4	122.24 (10)	C13—C14—C9	119.92 (10)
C6—C5—H5	118.9	C13—C14—H14	120.0
C4—C5—H5	118.9	C9—C14—H14	120.0
C5—C6—C1	116.93 (10)		
			
O2—N1—C1—C2	167.94 (11)	C7—O3—C8—O4	176.57 (11)
O1—N1—C1—C2	−12.17 (16)	C7—O3—C8—C9	−3.88 (15)
O2—N1—C1—C6	−12.66 (16)	O4—C8—C9—C14	0.2 (2)
O1—N1—C1—C6	167.23 (11)	O3—C8—C9—C14	−179.33 (10)
C6—C1—C2—C3	0.27 (18)	O4—C8—C9—C10	−179.16 (13)
N1—C1—C2—C3	179.63 (10)	O3—C8—C9—C10	1.35 (15)
C1—C2—C3—N2	−177.99 (11)	C7—N3—C10—C9	−1.60 (16)
C1—C2—C3—C4	0.75 (16)	C7—N3—C10—C11	179.43 (10)
N2—C3—C4—C5	177.25 (11)	C14—C9—C10—N3	−177.98 (10)
C2—C3—C4—C5	−1.41 (16)	C8—C9—C10—N3	1.34 (17)
N2—C3—C4—C7	−2.54 (18)	C14—C9—C10—C11	0.98 (17)
C2—C3—C4—C7	178.79 (10)	C8—C9—C10—C11	−179.70 (10)
C3—C4—C5—C6	1.12 (17)	N3—C10—C11—C12	177.86 (10)
C7—C4—C5—C6	−179.08 (11)	C9—C10—C11—C12	−1.14 (16)
C4—C5—C6—C1	−0.12 (17)	C10—C11—C12—C13	0.46 (18)
C2—C1—C6—C5	−0.60 (18)	C10—C11—C12—N4	−177.45 (10)
N1—C1—C6—C5	−179.96 (10)	O6—N4—C12—C11	170.38 (11)
C10—N3—C7—O3	−1.02 (16)	O5—N4—C12—C11	−8.20 (15)
C10—N3—C7—C4	179.81 (10)	O6—N4—C12—C13	−7.64 (16)
C8—O3—C7—N3	3.96 (17)	O5—N4—C12—C13	173.77 (11)
C8—O3—C7—C4	−176.79 (9)	C11—C12—C13—C14	0.44 (18)
C5—C4—C7—N3	−178.47 (11)	N4—C12—C13—C14	178.34 (10)
C3—C4—C7—N3	1.33 (18)	C12—C13—C14—C9	−0.62 (17)
C5—C4—C7—O3	2.27 (15)	C10—C9—C14—C13	−0.07 (18)
C3—C4—C7—O3	−177.93 (10)	C8—C9—C14—C13	−179.38 (11)

### Hydrogen-bond geometry (Å, º)

**Table:**

*D*—H···*A*	*D*—H	H···*A*	*D*···*A*	*D*—H···*A*
N2—H1*N*···N3	0.89 (1)	2.06 (1)	2.7124 (14)	130 (1)
N2—H2*N*···O1^i^	0.89 (1)	2.20 (1)	3.0691 (14)	166 (1)
C11—H11···O5^ii^	0.95	2.48	3.3248 (14)	149
C13—H13···O4^iii^	0.95	2.38	3.1778 (14)	141

Symmetry codes: (i) −*x*+1/2, *y*+1/2, −*z*−1/2; (ii) −*x*, −*y*+2, −*z*; (iii) −*x*+1/2, *y*+1/2, −*z*+1/2.
